# Origin of Yellow Fever

**Published:** 1880-07

**Authors:** 


### Editorial.

### ORIGIN OF YELLOW FEVER.

On the subject which heads this article we are sorry to find ourselves, for the present at least, in a hopeless minority. It may be that when the indigenous theory becomes more popular, many who now make no objection publicly to the importation doctrine, may come out boldly against it. Again, there are those who never think for themselves, and will therefore always be found with the majority, if they are not deceived as to which side has it.

The quarantinists, importationists, or contagionists, seem now to be in a muss, which may result in some good to the country. One party claims that the West Indies is the natural habitat of yellow fever, and that there alone it is endemic and indigenous. Another that it may originate in any tropical country or on vessels navigating their waters. A third insists that it always originates on marine vessels. Still another small class may be mentioned who hold that it is strictly a marine disease and cannot exist away from salt water.

In this multiplicity of views it is evident all cannot be correct, and we shall take the liberty of saying, probably none of them are true. This train of thought was suggested by, and this article commenced on receipt of a pamphlet entitled, "The Ship Origin of Yellow Fever with Comments on the Preliminary Report of the Havana Yellow Fever Commission; By Robert B. S. Hargis, Pensacola, Fla."

Dr. Chaille, President of the Havana Commission, and Dr. Hargis, passed a few shots at each other through the Pensacola Advance, a secular paper, which are recorded in the pamphlet before us. The scientific daggers thrust at each other were pointed and sharp, showing irreconcilable difference of opinion between them. They agree, however, on the utility of quarantine against yellow fever, and their spat originated in an attack made by Dr. Hargis on Dr. LeHardy's circular against increased quarantine power, in which Dr. Hargis incidentally makes a thrust at the Havana Commission in order to sustain his own view of the origin of the fever. From the pamphlet before us we are able to arrive at the true status of these two wings of the quarantinist or contagionists, and by their quotations, that of others. Contention between the advocates of these conflicting views must necessarily shake public confidence in their theories generally and thereby prove a blessing to the people. All contagionists agree that quarantine is necessary to protection, but the manner of conducting it is the difficult question. The ship originists have no use for a commission at Havana, because it is there upon the idea of Cuban origin, and Dr. Hargis shows a disposition to favor non-contagion and United States origin rather than approve the necessity of the Cuban Commission. Indeed he gives some very significant facts connected with the scourge of Philadelphia by yellow fever in 1793. He quotes as follows from the report of Dr. Charles Caldwell: "'No apartments being yet prepared for the use and accommodation of the medical assistants I was obliged to eat, drink and sleep (when indeed I was permitted to sleep) in the same room in which I ministered to the wants of the sick. And not only did I sleep in the same room with my patient, but also at times on the same bed." Dr. Caldwell was from this time an unbeliever in its contagiousness. He quotes further from a paper read before the Philadelphia Medical Society by Dr. Caldwell, as follows: "Yellow fever has but just disappeared, the miasm productive of it having been destroyed, as it always will be, by the occurrence of cold weather. My wish, therefore, sir," (addressing the chair) "is to hear from the gentlemen arguments in favor of its foreign origin, drawn from what they have learned by their recent intercouse with it, in the way of observation as men of science, and of experience as physicians engaged in the treatment of it." And again quoting from Caldwell's autobiography, alluding to this part of his paper he says: "This I knew would be gall and wormwood; because not a single individual who, as yet, had contended in the present debate, that yellow fever was an imported complaint had ever seen a case of it. They had all hurried into the country on its first appearance in Philadelphia and had now just but returned, to instruct the community, including those who had met it, contended with it, and studied it, as to the mystery of its origin."

To sustain his "ship origin" doctrine, however, Dr. Hargis shows his want of respect for such original thinkers and workers for truth in science, when he says, "Land malaria and filth on land are charged with the local production of yellow fever only by those who do not read and cannot observe." In the absence of proof for his doctrine strong and unwarrantable assertions are substituted for scientific facts. Would that physicians could always be governed in their opinions by undoubted facts, rather than theories of fine writers and speakers.

Dr. Hargis seems determined to drive his enemy to the unpopular doctrine of local land malarial origin unless he yield the point of his ship origin doctrine, when he intimates that the doctrine of *endemicity* leads us and the Board of health to the conclusion that yellow fever is one of the varieties of autumnal fever, as tertain is another. By endemicity he means the peculiar and constant tendency to appear in a particular country or among a particular people as was supposed by the Havana Commission, whose business it seems was to investigate that subject. We have then in Dr. Hargis an ally when we contend that yellow fever does not necessarily originate in the island of Cuba or in any other tropical land. If by this union of forces we can demolish the endemicites there will be no trouble in putting to rout the ship originists; for we will then have the money of the commission and numerous instances of undoubted land origin, both in and out of the tropics—by sea and by river side far in the interior. Then we say to Dr. LeHardy, neither despair, falter nor compromise on any point. The banner is unfurled—let it wave until the enemy shall have destroyed his power by internicine strife, and the scattered fragments will collect around the ensign of truth and common sense.

In previous contributions on the subject of yellow fever, its origin, etc., we have given facts and reasons that seem conclusive, proving its malarial origin, its noncontagionness, and that it originates not only on the United States coast, but many hundred miles from the sea, on rivers of sufficient size and length draining fertile land. These have also been faithfully compared with the reasons which influence the opinion of contagionists. It will not be amiss, however, to repeat some on each side of this question.

It is clear that the term "contagionists" is applicable to, and includes all who oppose the idea of indigenous, local malarial origin of yellow fever; for, if it be not contagious in the sense in which measles and small-pox are usually communicated, it certainly cannot be imported and communicated by cases of the disease and transported and made to prevail in the interior by the same means. Contagionists, then, have different reasons for and different modes of quarantine; yet all are for quarantine. The Cuban endemicists advocate the plan of United States' agencies at Havana and other tropical parts to apprize the authorities here of the sailing of any vessel with yellow fever subjects, so that quarantine laws may be enforced against them when attempting entrance to our ports. The ship originists of course require no such agencies, but a close watch upon all vessels hailing from tropical waters. The marine originists will of course accept the plans proposed by all for quarantine. Their views of origin makes it necessary to watch vessels from all countries where yellow fever may originate and our own coast likewise. All contagionists agree on the subject of quarantining the interior against public carriers from the coast in times of yellow fever prevalence.

Now, a great deal of expense, inconvenience and trouble generally, can be saved by taking what seems to be a common-sense, rational view of this subject, and prevent, as far as can be done, the cause of malaria at places where fever prevails, and if not successful, remove the inhabitants exposed at once on the appearance of fever. I know of no well authenticated facts at variance with the theory of the local land malarial origin of yellow fever. Every well attested case I have investigated occurring at a place where no malaria was produced can be accounted for by having visited a malarious neighborhood or by the malaria having been brought in a closed car, as was the case with Luffman on the Central Railroad of Georgia in 1876. Now take the thousands of cases carried into a non-malarious section where no one contracted the disease, and it will be seen that although the evidence is of a negative character, yet the amount is so overwhelming that it would seem to be convincing.

I first saw yellow fever about thirty-four years ago, three cases of which were treated at a farm residence in Middle Georgia. The subjects, three young men, probably contracted the disease at Panama, on their return from California, and all of them, I believe, were attacked before they arrived at home. One of them had a light attack, which resembled ordinary intermittent fever, except that it required longer and more vigorous treatment; another was more severely affected and had a case resembling the bilious remittent of that section, except a slightly deeper icterous hue and a little more persistence in the fever.

The worst case was of a malignant character, and although the young man lived for several days he had the violent symptoms of malignant yellow fever and died with black vomit.

The family and neighbors never having been instructed in the orthodox science of the present day, had no fears and were not affected in any way by their constant attendance upon these young men.

The next case I attended was in Atlanta, twenty-six years ago. Even then in a city like Atlanta, the people had not become sufficiently informed in popular medical science to entertain serious fears of danger, and the patient did not lack for the full number of attendants necessary for his comfort, besides visitors to the full extent allowable. The case was contracted in Augusta, Ga., was attacked and passed through the disease to a fatal termination with black vomit, without a trace of the disease being communicated to any who saw him.

Others cases not under my care were in Atlanta, I think the same year, from Savannah, without any damaging effect upon our people. Occasionally during epidemics at New Orleans and other places, since that time cases have been brought here with the same result.

Not until a few years past, have the people become sufficiently *enlightened* to fear any communication of the disease from one affected with it. It has been said that fear is a prominent factor in the production and fatal termination of yellow fever, but abundant proof against this view was afforded here in 1878, during the terrible scourge at Memphis that year. Some cases w re brought here, and some attacked after their arrival, all of whom died, according to my information, with unmistakable evidences of the disease. The nature of some of the cases were not known until free contact with the citizens had been had. One at any rate was taken from the train in the midst of a crowd, having been attacked twelve hours before, and, when it was ascertained that hundreds had been exposed to unmistakable yellow fever, some left the city, others raved and through the municipal authorities attempted to take revenge on the poor helpless sufferers by having the living ejected from the city into the woods, and conveying the dead to the grave without funeral. After all, notwithstanding the city was said to be in a filthy condition, not a trace of yellow fever was left among us.

If individual experiences were taken on this subject, much more convincing proof could doubtless be afforded than in the limited number of cases just adduced.

As to the theory of salt water being necessary to the production and spreading of the disease, the facts of two epidemics appearing in Augusta and in two successive years in Memphis, are sufficient to satisfy any reasonable person that there is no truth in it.

The reports made by the Georgia Board of Health in 1876, and that of the Yellow Fever Commission sent to New Orleans by the Surgeon-General of Marine Hospital Service, when closely analyzed, will convince any unbiased person that the fact of importation was very far from being proved by either. Both being put to work with preconceived views in favor of importation, as we have good reasons for believing, their reports show their bias, by expressions favoring that view without facts to warrant it.

At Savannah the Board of Health could not trace the disease to any one landing at that port affected with it, and yet there were expressions indicating a probability that it was brought from abroad in some way.

At Brunswick the disease attacked the Captain of a vessel in the harbor which had sometime during the year been in the tropics, and that without further statement of facts may evidently be taken as proof of importation, when all the facts, which were afterwards made known to me by a very reliable and intelligent physician of Brunswick, will leave no such impression. The Captain had been to New York and came overland by Atlanta to meet his vessel in Brunswick, and had lived in a boarding house or hotel in town from his arrival to the time of his attack. He evidently, therefore, contracted the disease in Brunswick. In another case the Board reported a seamstress as having been attacked the day after she had gone aboard a Spanish vessel lying in the harbor. The inference intended to be left was that the woman had contracted the disease in the ship, which no one familiar with the incubation of fever can possibly believe, when he reflects on the fact that only one day elapsed between the visit to the ship and the attack of fever.

The Yellow Fever Commission was sent to New Orleans with the view of tracing connection, if possible, between the epidemic of 1878 and the importation of the disease. The report of that body shows how far they were successful in this particular. Two cases of fever occurred in a ship's crew about the last of April or first of May, pronounced malarial fever at the time, but pronounced yellow fever by the Commission, from the history given them six months after the occurrence. Yellow fever was developed in another portion of the city from where these cases occurred, over one month afterwards. This was too long a period of incubation and the fever occurred too far from where the sick of the ship's crew were kept. After these facts were ascertained the Commission suddenly became in a great hury to leave, and said their time would not allow further investigation. The conclusion was, however, that although they could not trace any connection between the fever, where it broke out in the city, and the cases of the ship's crew or any other importation—it was nevertheless probably, they thought, brought in some undiscovered or unknown way. This report, under the facts developed, seems to indicate a preconceived bias in favor of importation.

The idea of importation and contagion is easily impressed and maintained in a panicky community; and as ships are all the time landing on the coast and boats and railroads carrying sick and well refugees from infected places to almost all others, it is an easy matter to fix the conclusion in the minds of the people that the fever is contracted by coming in contact with cases of the disease.

For Dr. Hargis' especial benefit we note the fact that even when the crew of a ship have contracted the disease on board or otherwise, numerous instances of failure to spread on shore after landing can no doubt be called to mind by himself. Taken, therefore, altogether, the failures to spread from the presence of cases of the disease, are larger, in proportion to the larger number of non-malarious places where the disease is carried.

